# “Mental maps”: Between memorial transcription and symbolic projection

**DOI:** 10.3389/fpsyg.2023.1142238

**Published:** 2023-03-28

**Authors:** Bernard Guelton

**Affiliations:** Institut ACTE, Université Paris 1 Panthéon Sorbonne, Paris, France

**Keywords:** mental map, mind map, cognitive map, graphic transcription, cognitive graph

## Abstract

“The mental map” is a concept that has been used and defined in numerous ways. The cognitive map, and the concept map–also known as the “heuristic” or “mind” map–are the two distinct contextual meanings covered by the term mental map in the present article. In the mental map domain, the first major field of study is geography, spatial cognition, and neurophysiology and it aims to understand how the route taken by a subject (or a set of subjects) in space leads to memorization and internal representation(s). In general, the externalization of these representations takes the form of drawings, positioning in a graph, or oral/textual narratives, but it is primarily reflected as a behavior in space that can be recorded as tracking items. A second field of study, one which is geared more toward exploratory and combinatorial uses, is the concept (also heuristic or mind) map which consists in organizing notions, concepts, and information in the form of tree graphs or graphs that can be used to produce diagrams and flowcharts. The aim is projective, for clarification and discovery purposes or for data organization and visualization. To date, very few studies in the literature have examined the similar, overlapping and oppositional features in what is broadly referred to as “representation(s) of space” and “space(s) of representation.” How can we better apprehend the complex notion of “mental map?” The question of memorial transcription? Of “symbolic projection?” Can we identify meeting points between these two polarities and, if possible, a continuum? Through the notion of cognitive graph, recent advances in the understanding of brain mechanisms enable us to approach the distinctions between cognitive map and conceptual map as an articulated and continuous whole.

## 1. Introduction

Although mental (mind) maps, concept maps and cognitive maps (Chauvin, 2010) are sometimes grouped together under the umbrella term “semantic maps,” the notion of “mental maps” will be considered here according to two different meanings and uses: (1) the internal representation of a traversed space (**cognitive map**), and (2) the representation of a set of entities or concepts (**concept map, mind map**). It must be noted from the outset that the English term *mental map* does not usually cover the same scope as the French term *carte mentale* [mental map] and is mostly limited to the meanings of concept map or *carte heuristique* [heuristic map]. Here, the term “memorial transcription” is understood to mean the translation and recording on a graphic medium of elements that are mentally present, whether it is the memory of a traversed space or of a set of information or concepts to be arranged, and the term “symbolic projection” is understood as the externalization, through signs or symbols on a graphic medium, of the two types of aforementioned representations–that of a traversed space or of a set of entities or concepts (Illustrations 1–3).^1^

**ILLUSTRATION 1:**
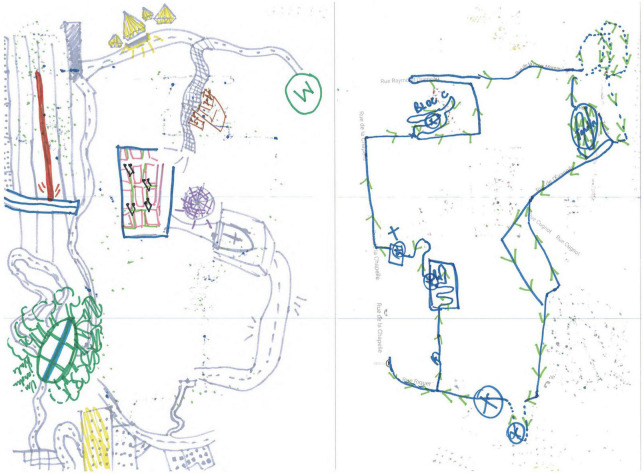
Examples of urban cognitive map drawings. From the research project: Situated media and shared mobilities: mental, instrumental and shared cartographies 2017–2019.

**ILLUSTRATION 2:**
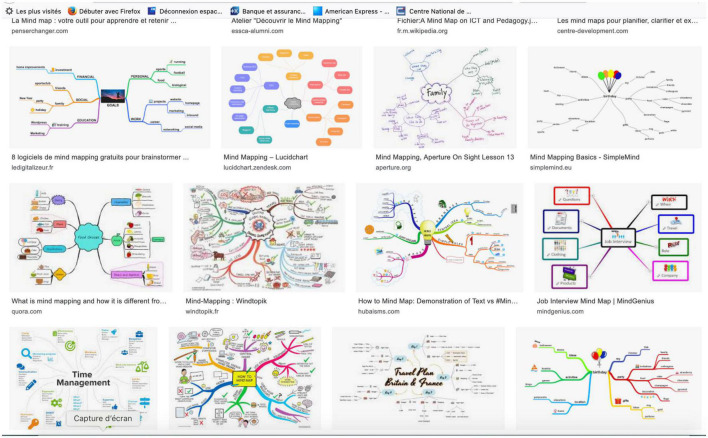
Examples of concept maps (mind maps).

**ILLUSTRATION 3:**
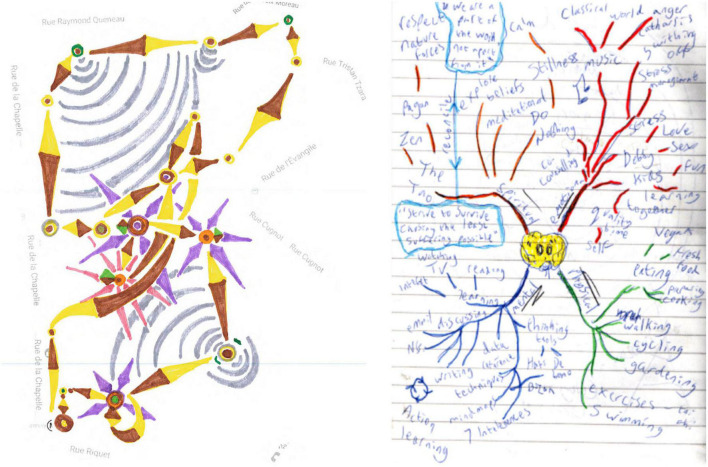
Restitution of an urban space **(left)** and Example of a mind map **(right)**.

Whether we are dealing with the mental representation of a traversed space, or the representation of a set of entities or concepts, each of these representations is the object of an internal understanding before it is transcribed externally on a medium, in other words, both require an *externalization process*. The former is subjected mainly to a memorial process (a previous experience in the physical world), and the latter projects entities that need not necessarily be pre-existing, except as prior internal representations (information or concepts). In actual fact, it is a dual, interactive process in which the transcription process interacts with the subject’s memorization and internal representation processes (Massironi, 2004; Shimojima and Katagiri, 2008; Ware, 2020^2^). In other words, the drawing is produced from elements present in the subject’s internal memory, but this memory is reconstructed through this graphic representation. Working memory and its role in drawing composition in relation to long-term memory is a very interesting question but, to our knowledge, one which is rarely discussed. Another issue that should also be considered is that of episodic memory (lived events and experiences) and the memorization of cognitive maps. In both cases, the two types of maps determine the individualized entities and the relationships between them. Also, in both cases, there is memorization and projection, in other words, elements to be retained are selected and “operations” between entities are set up. The former is subjected to the body memory of a route in a space and calls upon spatial cognition, and the latter is directly dependent on an abstract spatial representation, a more or less conventional “blank page” in which entities and relations between them can be located.

The article will be organized as follows. (In the section “2. Materials and methods”), we will first present an overview of the knowledge involved in the proposed questioning, followed by some examples of urban cognitive maps (Illustrations 1, 4) and two stop motion videos showing the different phases of producing a sketch map (Ilustrations 5, 6). An overview of certain characteristics of cognitive maps will be provided, and the semantics of graphs will be briefly covered in order to put the notion of “symbolic projection” into context. Some questions on memorization from a cognitive map and concept map perspective will extend this aspect (for the context of “Memorial transcription”). The final discussion will conclude by addressing recent advances in brain neurophysiology proposing the notion of cognitive graphs as a common substrate to link cognitive maps and concept maps.

**ILLUSTRATION 4:**
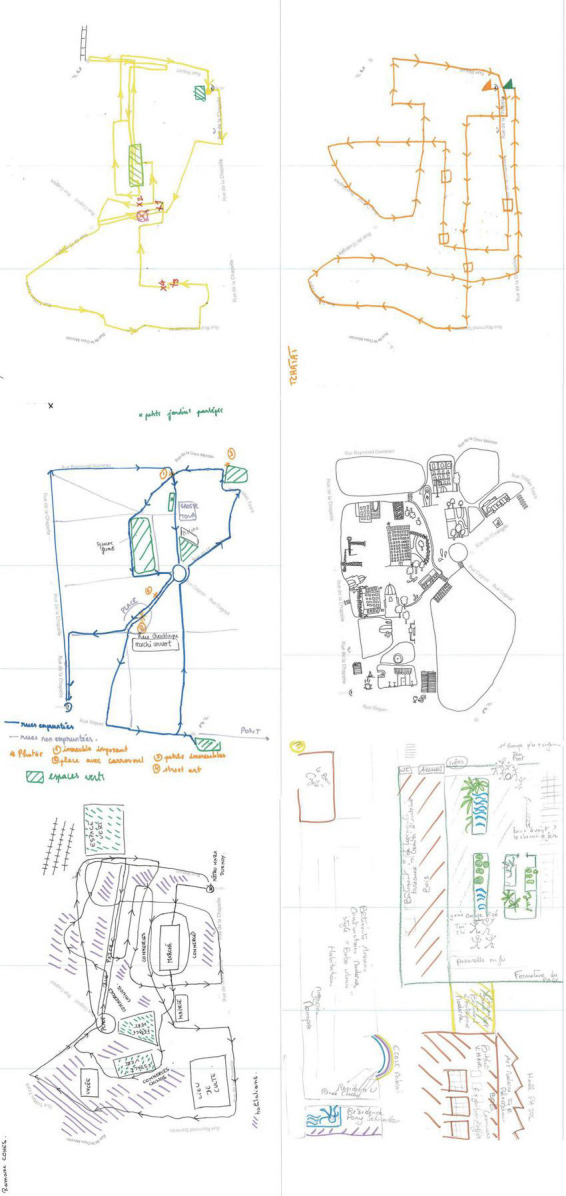
Different strategies for urban sketch maps.

**ILLUSTRATION 5:**
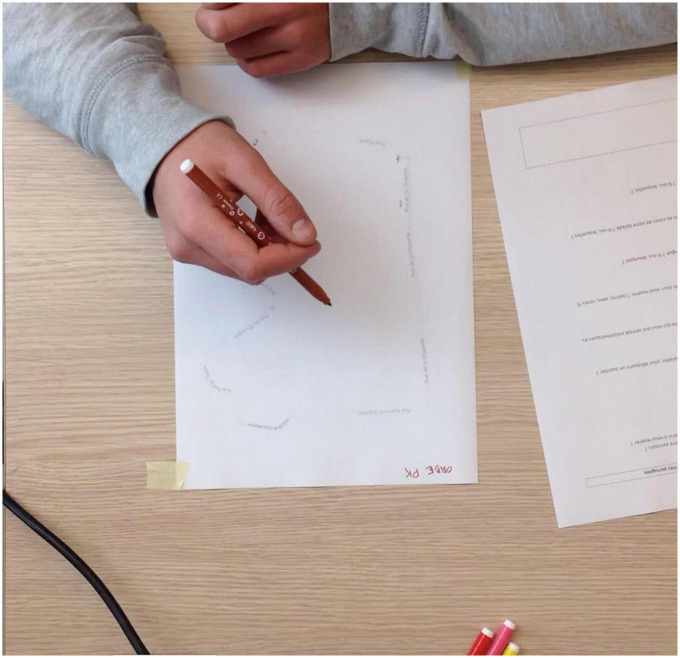
First stop motion video. https://vimeo.com/428121651.

**ILLUSTRATION 6:**
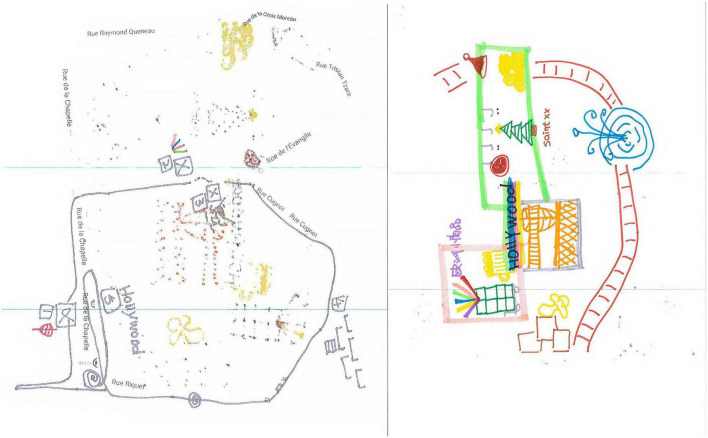
Second video: Two successive drawings on the front and back of the same sheet of paper. https://vimeo.com/428128358.

## 2. Materials and methods

### 2.1. Diverse knowledge

The diversity of the literature and experimental sources that attempt to shed light on the construction of these two types of maps is very broad and diverse. It spans neurophysiology (Tolman, 1948–who was the first to use and mediate the notion of the cognitive map–Kosslyn, 1990; Wills et al., 2010), psychology (Arnheim, 1969; Shepard and Metzler, 1971; Johnson-Laird, 1983), cognitive psychology (Tversky and Taylor, 1992, 1996; Kitchin and Bladen, 2002; Wakabayashi and Ishkawa, 2011; Tversky et al., 2012), graphic mapping (Bertin, 1977), and data visualization (Ware, 2020). All of these fields relate in varying degrees to the topics discussed. “Visual thinking” is explicitly mentioned by Arnheim (1969), National Research Council (2006), and study by the Ishikawa (2016), in which the importance of visual thinking, which is viewed as essential, is to be promoted. These few authors, of course, represent only a very small part of the available resources that are constantly being developed. A cognitive map refers to an internal representation specific to each human or animal individual (or group of individuals or animals) that is constructed during or after movement in a physical or virtual space. The cognitive map notion was introduced by Tolman (1948) and has since been studied and debated by a number of authors. A series of experiments has shown the capability of organisms to use three sources of information during their movement processes: dimension, orientation and the subject’s own movement. In Arnheim’s (1969) book *Visual Thinking* (published in 1969 by the *University of California Press*), the author states that the old dichotomy between seeing and thinking, perceiving and reasoning would be false and misleading, showing that the fundamental process of seeing encompasses the typical mechanisms of reasoning. The images of thoughts, the forms of concepts, and the nature of abstraction are some of the essential points of this seminal work. Many articles and reflections have since updated this questioning, giving it experimental bases. For example, see the recent work by Ware (2020) entitled *Information visualization*. The existence of mental images, of their materiality (rotation time, competition with a physical stimulus), have been demonstrated by Kosslyn’s (1990) widely recognized experiments (1987, 1994, 1993, 2003) and Shepard (1985). The concordance between the rotation time of a physical object and that of its mental representation has shown, along with other experiments, the “materiality” of mental images. As for graphic transcription, Bertin’s (1977) work, *La graphique et le traitement graphique de l’information*, published in 1977, is universally recognized as a seminal work on the coding of graphic information and is an essential basis for more recent developments in data visualization. The specificity of graphics, its bases, the image variables, and image separation variables are some essential points of visual information processing that are still operational today. However, it is the recent developments in neuroscience and the study of the human and animal brain that are now making decisive contributions to advances in our understanding of what a cognitive map is by linking two notions that were once opposed at the basis of this research. Among the growing number of studies on the subject, let us mention the study by Peer et al. (2021), which highlights the importance of cognitive graphs for spatial knowledge based on the study of the functioning of cerebral areas, while showing that cognitive maps and graphs can function simultaneously or separately.

The considerable diversity of the knowledge involved is evident. The issues of mental imagery, whether in an essentially memorial form relating to the exploration of a physical space, or as an organized representation of a set of information or concepts, are concerned by the advances and reflections of the authors who have just been cited. We believe that the similar, overlapping and oppositional features between “representation(s) of space” and “space(s) of representation” have rarely been examined and as such, we set out to identify some meeting points, and possibly a continuum, between these two uses. We could start by dividing the question into three underlying questions. In the drawing of an urban mental map, (1) How does the memorial process take place? (2) How is the symbolic process of drawing carried out? And (3) How do the two processes interact? As with many “simple” questions, these are big issues that require more targeted exploration, especially with the addition of a second context of Mind Mapping, concept maps or concept networks, as we are doing here. This second domain raises even more directly the matter of a symbolic transcription that is considered here under the terms of symbolic *projection*. We use the term “projection” because, in addition to the restitutive aim of drawing an urban mental map, this involves the projection of a space for the arrangement of notions or concepts that have already been identified or, on the contrary, whose perimeter has yet to be determined.

### 2.2. Examples of urban cognitive maps

The examples of urban cognitive maps will be shown here using two types of examples: (1) the sketch of a brief typology of urban cognitive maps, and (2) two stop motion videos showing three sketch maps being realized.

### 2.3. Two stop motion videos of sketch maps

Two stop motion videos will serve as the basis of our reflection and allow us to explore these issues.^3^

https://vimeo.com/428121651.

https://vimeo.com/428128358.

http://fictions-et-interactions.net/recherche/.

These two videos show a drawing being realized from the memory of a 1-h walk in a Parisian neighborhood. The first video shows the hesitations of the draftsperson, who seems to be wondering about how to proceed, for example, where and how to draw the lines, how long and in which direction should they be, how to situate different traversed zones in the neighborhood, and how and where to represent the landmark points. The issue of externalization in the creation of this mental map is fundamental. Two types of representation are involved: *allocentric representation*, for the capability to globally remember a set of elements situated in relation to one another, and *egocentric representation* for the capability to memorize the succession of steps accomplished along the route. For us, the second video is quite surprising and particularly relevant for this study, as after the draftsperson has produced an initial drawing using the neighborhood’s predetermined outline, she suddenly turns over the sheet of paper to position several different types of symbol, for example, of a simple square of color or a detailed fountain.

#### 2.3.1. Analysis of the first video

This first 3-min video was broken down into around fifteen successive steps. We were struck by the amount of hovering over the drawing by both pencil and hand, with parts of the drawing being retraced without actually following through with the drawing. It would seem that approximately half of this drawing sequence is occupied by gestures that sketch out tracing movements but without following through. This drawing, like most urban mental map drawings, contrasts drawings of more or less symbolized landmark points and linear tracings. Landmark points and road tracings are the two key components in the production of this type of drawing. This is a common observation. Liaisons of crossing points, connections between landmarks, and the orientation of directions form an immediate extension to the drawing of main roads and delimitations of the whole territory. In this first video, the district’s general delimitation is the subject of the drawing’s first sketch. There are some written indications specifying the names of main sites, or photographs that the draftsperson had been asked to take during their exploration of the district.

#### 2.3.2. Second video: Two successive drawings

While the first drawing is restricted to a continuous traced line correlated to the map outline and has a few landmark points, as is usually the case, in the second drawing the draftsperson has freely made arrangements with many colors, sectors and symbols that are more or less abstract or clearly representative. This is no longer a matter of trying to transcribe a more or less allographic or egocentric representation, it is the spontaneous arrangement of about fifteen symbolic elements in the space of the sheet of paper. The arrangement is both abstract and figurative, hierarchical in terms of the dimensions of the represented elements, and topographical as regards positioning and inclusions.

### 2.4. Urban cognitive maps

In the brief presentation of cognitive maps, it was mentioned that a series of experiments revealed the capability for organisms to use three different sources of information during their movement processes–dimension, orientation, and self-motion–through three mechanisms: trajectory integration, orientation relative to landmark points, and geometric calculation based on dimensionality in space. Memorization and then externalization *via* a graphic transcription show diverse strategies which have just been illustrated. They are characterized by the fact that they are “collages” reflecting distortions and omissions.

#### 2.4.1. Collages

Through these examples, we can observe a diversity of strategies used to externalize the memory of an urban space. It is important to bear in mind that there are, at the same time, two elements at play: the subject’s internal representation proper, and the capability that varies with each individual to translate this into a graphic representation. The graphic representation in most cases provides landmark points and roads, but it is a representation which is not constructed in a homogeneous and continuous space, but rather as a patchwork. In Tversky (1993) we note this fundamental dimension of a collage that distorts the reality of the physical space and omits parts of it. Urban map memorization is not a continuous encoding of information; it distorts, regularizes and omits information, but retains an operational relevance to the reality of physical space.

#### 2.4.2. Distortions

Distortions consist of over- or under-estimating some dimensions over others. They can be the size of the areas identified and traversed, the distance between particular landmarks, or any changes in direction made during the journey, changes that are often simplified to orthogonal angulations. More elaborately, the hierarchical relationships between distances, landmarks or sub-neighborhoods are distorted. Once again, these distortions are both specific to the memories present in the mind and also the result of the simplifications made by the graphic transcription.

#### 2.4.3. Omissions

Ultimately, only the objectives of memory representation matter. Is the objective to find a particular location or address? To point it out to another person? To identify the journey to be made between a place of residence or a means of transport? To identify pleasant areas to walk through or those to avoid? In these different situations, useful information will be valued and other items forgotten. These characteristics resulting from collage, distortion or omission are just as present when successively reconstructing a route in a previously unknown space, or representing geographical data known to all, such as the map of France or the distances between the cities of Marseille, Strasbourg or Nantes, for example. The notion of “map” for cognitive maps has been widely debated. As Cauvin (1999) underlines, following Kitchin (1994), – “they are explicit, i.e., they are maps, – they are analogical, i.e., they are like maps, – they are metaphors, i.e., they function as if they were maps, – they are hypothetical constructions and are in fact a practical fiction.”

Of course, these distortions and omissions should not be understood as “mistakes” but as convenient ways to memorize and orient oneself in space.

### 2.5. Graph semantics in brief

#### 2.5.1. Internal data, external data, and graphical entities?

Concept maps (also called mind maps^4^) were made popular by Buzan and Buzan (2003). They are essentially designed as a drawing of a radiating diagram organizing a number of ideas from a central concept. As previously mentioned, mind maps, (unlike cognitive maps), do not involve the immediate memory of moving the body through a physical space and then having to externalize a drawn representation of it. The experience occurs directly in the construction of a graphic representation. This is, and will remain, a fundamental difference between what is referred to here as concept maps (mind maps) and cognitive maps. That being said, the concept map does call upon “pre-existing” elements, the constituents of which require further clarification. Moreover, the body experience conditions all representations of space, whether this is a spatial recollection and/or a graphic transcription. The question is then to try and better define what exists prior to the graphic realization of the mental map as internal or external data to the subject, whether it is the social universe or the physical world. The term “data” means that existing information precedes the construction of a concept map, whereas the term “entity” represents an element in the drawing that is capable of representing existing data or data that is free of all external references.

#### 2.5.2. Graphs

The mental map can be represented in the form of a graph, i.e., a diagram constructed from a set of nodes connected by edges that represent relationships between the nodes. It is defined as follows (Archambault and Purchase, 2013): “A graph consists of a set of nodes V, together with a set of edges E, which represent relationships between nodes. Graph drawing is the process of assigning coordinates to the nodes so that a node-link representation of the graph can be depicted in a two-dimensional plane. Dynamic graph drawing deals with graphs that evolve over time, whereby the graph structure changes as nodes and edges are added or removed.” One may wonder whether the elementary graphical organization of *nodes* and *lines* in mind maps, and of *landmarks and roads* in cognitive maps can constitute, if not a common cognitive background then at least an analogous representational convenience. This would seem to be the view of Archambault and Purchase (2013). But it is more recent evidence that confirms the mixed nature of cognitive maps and graphs (Schafer and Schiller, 2018; Timothy et al., 2018; Peer et al., 2021). As mentioned earlier, nodes in a mental map may represent data that already (or potentially) exists in the draftsperson’s mind, or they may simply be points in an abstract relationships network. Lines may therefore represent particular types of relationships to be specified or inventoried between nodes. For cognitive maps, the same graphical nodes and lines structure represent landmark points and roads. The landmark points represent significant elements or events (typically visual) that have emerged along the route through the physical space, while roads enable these landmark points to be connected (most often partially).

##### 2.5.2.1. Distances and topography between nodes

The distance between nodes can represent distance or proximity between two concepts or between two landmark points. In addition to this fundamental characteristic, the orientation of the medium determines the upper or lower, right, or left zones of the sheet (or screen). This is a minimum topography that characterizes locations of nodes and correlatively the relationships between them.

##### 2.5.2.2. Orientations and directions

###### 2.5.2.2.1. Reading direction

Independently of a theoretical understanding of graphs, an implicit reading direction (European languages) favors reading the graph from left to right and from top to bottom. These left/right and top/bottom orientations are likely to determine a succession in the reading of nodes and lines and thus an underlying interpretation of the graph.

###### 2.5.2.2.2. Angular directions

It is worthwhile considering directions, i.e., the angulations that result in the junction of nodes, when comparing spatiotemporal or semantic graphs. In the spatiotemporal graph, a succession of orientation and direction changes are added to the landmark points, and to their distancing are added a succession of orientation and direction changes, which leads to a fundamental determination in the memorization of a route. In other words, while angulations may result from a purely conventional neutral regime in semantic graphs, these angulations are no longer so in the context of cognitive maps.

##### 2.5.2.3. Arrowing

These angulations can depend on the orientation of the graphical medium. The overall orientation of the graph complicates these initial considerations, depending on whether the top/bottom and right/left orientations of the medium are considered to be agreed and fixed, for example, or whether an arrowing option is added to the edges or lines. The latter is likely to orient the graph in a specific or autonomous way with respect to the conventions in the medium’s layout and thus a neutral or basic spatiotemporal framework.

##### 2.5.2.4. Topological properties

In graph theory, a graph can be oriented or non-oriented (arrowed or not) and, from the topological perspective there are three main graph categories: structured, arbitrary and multipolar. There are four kinds of structured graphs: homogeneous, hierarchical, cyclic, and polar.^5^ Spatial topological analysis makes it possible to analyze the spatial relations between the objects with the notions of continuity, limit, neighborhood, inclusion, intersection, connectedness, connectivity, nodality, and accessibility.^6^ According to Egenhofer and Franzosa (2007), the relations between interior, enclosure, and boundaries are essential for spatial topological configurations and can be extended to the following situations: equal-non-equal, inside-outside, intersections. In terms of both cognitive and concept maps, these topological relations can be significant and important depending on whether or not they preserve some of these properties according to the changes brought about by continuous deformations “without breaks or tears,” as is customary when characterizing topological relations.

##### 2.5.2.5. Constraints and strategies

The constraints that govern the distances and relations between nodes are relative to the requirements of the person (or algorithm) who is drawing the graph. For semantic graphs, the principal layout requirement is for homogeneity and coherence of the whole. However, such coherence is not created from the outset, but achieved by trial and error within a predetermined framework. A spatiotemporal graph, which is the memorial re-transcription of an urban route, is most often a collage of more or less incongruent elements, with distortions and omissions in relation to what would be represented by a geographical map. (Strategies vary from one subject to another, some giving priority to the arrangement of landmark points and then to the roads or tracings linking these landmarks. Or conversely, it is the roads and tracings that determine the framework for the placement of landmarks. Of course, these are most often mixed strategies with preferred patterns). Since it is the memorial re-transcription that takes precedence, it is not uncommon to see the drawing spill over the frame of the sheet of paper, or display sudden breaks in scale to make it fit the medium.

##### 2.5.2.6. Semantic relations

Now may be an opportune moment to inventory, beyond a network of nodes and lines, other types of more complex graphic representations. It will be possible to: individuate categories, aggregate entities, and relativize distances between entities. These elements will be directly significant when there is sufficient congruence between the graphic space and the relationships to be established. Three structuring levels can be considered: (1) *page properties* (positioning and proximity, centrality, horizontality versus top-bottom, verticality versus left-right), (2) *forms of markings* (points, lines, arrows, boxes, zones, and symbols) which are likely to produce as many meanings, and (3) *visual expressions of these meanings*: individuations, types, orders, relationships, correspondences, continuums, hierarchies that are similar to those that exist in everyday language and particularly in the models created when people design the world around them. “The designed world is a diagram” (Tversky, 2011).

##### 2.5.2.7. Operations

The different types of relationships, page properties, forms of marking and visual expression of these meanings (listed above) authorize so many operations that are enabled with much more freedom for concept maps than for cognitive maps which are subject to more restrictive regulations. It is the representation of a route in space that combines egocentric and allocentric memorization, taking into account the constrained space of the sheet of paper (or the graphics tablet) and the subject’s ability to draw. However, as we saw in the example of the second video, for some people, the cognitive map can be easily converted into a configuration similar to the concept map. By operations, we mean all the actions permitted by the changes to relations, page properties and forms of markings, with the most obvious being to move an element or a set of elements, reduce or enlarge their dimensions, reduce or extend an arrow between two entities and multiply connections. In addition to these more or less intuitive operations, which are free of established models, there are of course widely recognized diagrammatic forms that are suitable for specific representation types that formalize results such as bars, histograms, point clouds, sectors, and polar projections.^7^ Categorization, order, intervals, ratios and proportions form categories to produce and communicate meaning in a graph. However, more elaborate forms such as the Venn diagram or the semiotic square look beyond the quantification and formalization of existing data to operations of discovery in spaces of concepts or abstract notions.

##### 2.5.2.8. Visual thinking

“The abstract thought has roots in the spatial world. Abstractions are expressed in the way things are arranged in the world, as well as in the way people speak and gesture. Arrangements on the page should be best when they are congruent, that is, when the abstract concept matches the spatial concept. Congruent matches can be revealed in people’s performances and preferences. Congruence is considered here for visual representations of continuum and category. Congruently configuring a continuum concept, frequency with a visual continuum variable and configuring a category concept, inclusion with a visual categorical variable, all are preferred and produce better results than the reverse situation (Cox et al., 2012). There is a continuity between the body’s experience of space and abstract entities or concepts representations. In other words, the abstract representation has its roots in the perceptual experience of space. That is, (1) space of the body, (2) space around the body, (3) space of navigation, (4) space of the graph (Tversky, 2003).”

### 2.6. Memorial process, sequentialization

“Cognitive tools increase the capacity of the mind in 2 main ways. They reduce the memory load by externalizing memory. They also reduce the memory load by allowing operations and calculations to be done externally, rather than on internal objects, and by enabling immediate external productions (Tversky, 2000).”

Tulving (1983) by developing different models for episodic memory is one of the first to have conceptualized episodic memory, that is, the memory of events experienced personally. Episodic memory is essential for orientation in time and space and is also known as the What-Where-When memory (Griffiths et al., 1999) or the What-Where-When (WWW) criteria (Suddendorf and Busby, 2003). This formulation is well-suited to the process at work in cognitive maps. This emblematic aspect of What-Where-When memory for cognitive maps could perhaps be transformed into What-Where-How for concept maps, but it should not be overlooked that the “how” is central to the graphic transcription of both types of maps. Tulving’s (1985) decisive contribution consists in the integration with episodic memory (in other words, the mental capability to reconstruct past events) of the conception of a “Mental Time Travel” (MTT), which not only makes it possible to reconstruct the past, but also to imagine possible scenarios in the future. Magnetic Resonance Imaging (MRI) studies have shown that the same region of the brain is used for both remembering the past and for imagining a similar event in the future, which shows that past memory is also used and activated when future projections are made.

#### 2.6.1. Urban cognitive map

##### 2.6.1.1. Stages of walking

As previously mentioned, the sources of information for memorization in the construction of a cognitive map have been identified by a series of experiments revealing that organisms have the capacity to use three sources of information during their movement processes–dimension, orientation and self-motion–through three mechanisms: (1) trajectory integration (based on proprioceptive data related to body movement, see Etienne and Jeffery, 2004), (2) orientation relative to landmark points (salient features in the environment), and (3) geometric computation based on dimensionality in space (Tversky, 2003, 2004). Although they can operate separately (see Etienne and Jeffery, 2004; Cheng and Newcombe, 2005), they can also work together to construct a posteriori map that integrate three different cognitive scales: body space, (embodied and situated cognition, egocentric perception), the space around the body (situated cognition, egocentric and allocentric perceptions), and movement space (allocentric perception and sometimes extended cognition) (see Berthoz, 1997). More commonly, the identification of numerous landmark points, of distances between these landmark points and of changes in orientation made during the journey, are the basic elements of a memorization of space. An egocentric or allocentric representation, most often a mixed one, helps to integrate this memorization in an organized way. For Colin Ware, two types of information are important in terms of strategy.

(1) People use a variety of strategies to navigate their environment (Ekstrom et al., 2018). The types of knowledge used are varied and include the following: declarative knowledge, which is non-spatial knowledge of locations and landmarks; procedural knowledge about the sequence of turns and methods used to get from one place to another; topological knowledge about the location of navigation routes and how they are interconnected; and spatial knowledge about the arrangement of locations in space, as in a map view (2). In one of the earliest influential theories, Seigel and White (1975) proposed that knowledge is acquired sequentially. First, declarative information about key landmarks is learned; second, procedural knowledge about routes from one place to another is developed; third, a cognitive spatial map is formed. Now, this view has been discredited (e.g., Zhang et al., 2014), and it is now plain that all three types of information, as well as conceptual and navigational strategies operate early on, when children are learning to navigate their environment. The most important function of memory is not to retrieve past events but to predict future ones (Clark, 2013).

##### 2.6.1.2. Drawing stages

The drawing stage that follows spatial exploration will most often be based on these same elements: landmarks, relationships between landmarks, distancing, and area boundaries. Different strategies have been exemplified above, with what we call refer to here “traces and orientations, landmarks, sectorizations, and mixed strategies.” The graphic transcriptions may vary considerably from one individual to another while remaining within a general framework that associates landmarks and tracings or nodes and lines in the manner of graphs. The outer limits to the area traversed does not seem to be an issue related to the organization of landmarks and lines, and it can be observed in the first video which documented that laying out a “border” or limit to the area to be represented is one of the draftstperson’s first tasks. In this case, in terms of memorization, it can be seen that this is not a matter of the restitution of a series of steps chronologically, but rather the organization of an allocentric framework from the very outset.

Through observing the construction dynamics of cognitive map drawings of around 40 people,^8^ it can be seen that an additional strategy can be added to that of the classical overall outline–a central zone of the drawing from which numerous directions emerge that will determine the general framework. A third strategy can also be observed–an approach that ignores an outline or a center, but proceeds by directly aggregating elements identified in the urban route. These elements are then grouped by zones, in “packages” or linear branches. Most of the time, the description of cognitive map drawings as a patchwork of more or less heterogeneous elements is true. The breaks in scale disrupt the homogeneity and continuity of the two geometric dimensions of the sheet of paper, leaving the draftsperson in a state of uncertainty as to the choices to be made.

#### 2.6.2. Concept map

##### 2.6.2.1. Distinctive features for memory in concept maps

While working memory and episodic memory coupled with semantic memory seem to be decisive for cognitive maps, the same is probably not true for concept maps in which episodic memory should not be as central as it is for cognitive maps. In contrast, here, semantic memory should play a predominant role to the extent that we can sometimes speak of semantic maps for a set covering both types of maps (Chauvin, 2010). Nevertheless, it should be pointed out that although concept maps do not in themselves deal with spatial data–as the article by Peer et al. (2021) seems to suggest–their graphic formatting is, as with cognitive maps, based on spatial formatting.

##### 2.6.2.2. Trial and error

Concept maps can most often take the form of graphs (structured, arbitrary and multipolar). As a result, the graphical and semantic properties that have been mentioned–distances between nodes, directions, topological properties, semantic relations and the resulting operations–are fully applicable. All of these elements are most often the subject of trial and error, of deletions and restarts, in an attempt to find an adequate form at both the global and local level, as these two levels may appear contradictory or inconsistent. Moreover, what was considered at one point in the graphic treatment as a “mistake” may eventually prove to be fruitful by allowing the discovery of new and unexpected relationships. These tentative steps, trials and errors are of course present in what has been shown in a map.

#### 2.6.3. Comparisons

In view of the most widely shared knowledge and distinctions about memory (the distinctions between different types of memory such as explicit and implicit, declarative and procedural, episodic and semantic), an attempt to investigate memorial processes in the transcription of a cognitive map and the transcription of a concept map is proving to be most difficult. First of all, it seems evident that the memorial process is of a completely different complexity for the drawing that memorizes a route in a physical space than for the drawing that attempts to visualize concepts or data. One may ask why this is the case. On the one hand it is because the cognitive map most often requires two types of perspective to be coordinated: egocentric (“on the way,” the memory of the route’s different stages) and allocentric (“on the fly,” the one that deduces an overall and joint organization of the route’s main elements). While some subjects are more inclined toward one type of perspective over another, it is generally the case of a mixed perspective. In the cognitive maps documented here, certain tendencies can clearly be seen. That being said, memory drawing on paper is often consistent with an allocentric representation. On the other hand, it is because two quite distinct memorial processes need to be coordinated, (1) a route carried out in space and (2) a series of successive actions necessary for the realization of the drawing itself. Added to this is a memorization in which it is not possible to go back to the movement carried out. However, in drawing, the succession of reported events can be corrected.

Moreover, it is a case of organizing, in the drawing of a cognitive map, a strategy that spares the memorization of a series of graphic actions and, at the same time, the future programming of a series of actions to be carried out–the whole–in a manner consistent with the memory of the actions conducted in the physical space. The major difficulty, the “black box” we know almost nothing about, is the connections and interactions between these two memory processes. In contrast, the realization of a concept map, although it also calls upon a memorization process, primarily focuses on the construction of the drawing and does not have the same complexity as a dual translation.

### 2.7. Cognitive and neuronal implications

Recent studies in brain neurophysiology, in cognitive sciences and more theoretical analyses report in-depth research that details the notion of cognitive map, cognitive graph, their close interrelations, or the predominance of one or the other according to the contexts considered. They allow us to understand and explain the common structure in the seminal opposition discussed in our article: that between cognitive map and conceptual map in the common name of mind map. The question of a common substratum from the point of view of the functioning of the cerebral areas is now widely explored. In this last part, we review and summarize some researches and hypotheses in neurophysiology concerning a general structure combining cognitive maps and cognitive graphs, even going so far as to consider a coding of social cognition. Thus, the cognitive graph allows us to understand the continuity between the cognitive map and the conceptual map. We then discuss three behavioral studies that consider the prevalence of cognitive graphs for spatial orientation. Finally, we recall that older, more theoretical studies such as that of Meilinger (2008) already proposed a synthesis of cognitive maps and graphs in the context of the study of behavior and orientation in space.

For Peer et al. (2021) humans and animals use mental representations of the structure of the world to navigate. Rather than Euclidean^9^ cognitive maps, they point to alternative theories suggesting that they are cognitive graphs composed of places connected by paths. Rather than being competing hypotheses, cognitive maps and cognitive graphs may coexist in the same individuals, with their availability and use depending on the characteristics of the environment and the requirements of navigation. Cognitive maps and cognitive graphs are instantiated by partially distinct, but partially overlapping neural systems in hippocampal formation, frontal lobes, and scene-selective cortical regions. Both representational systems can presumably support abstract thinking; Euclidean maps are suited to representing content varying along continuous dimensions, whereas cognitive graphs are suited to representing state transitions and discrete associations between items. The discovery of place cells in the rodent hippocampus has provided some of the evidence for a spatial code in the brain. One study (Howard et al., 2014) showed that activity in the anterior hippocampus and entorhinal cortex was related to Euclidean distance, while activity in the posterior hippocampus was related to path distance. The Peer et al. (2021) study successively presents evidence for Euclidean spatial representations and evidence for graph-based spatial representations. Documenting several neuroimaging studies, they distinguish between the different brain areas engaged depending on whether the system is a cognitive map system (activation of the hippocampus, entorhinal cortex, and medial and orbitofrontal prefrontal cortex) or a cognitive graph system (activation of the retrosplenial complex, hippocampus, and medial and orbitofrontal prefrontal cortex).

Timothy et al. (2018) document recent work and describe neural parallels between spatial and non-spatial behaviors that have revived the notion of systematic organization of knowledge across multiple domains. They describe with many authors the discovery of specialized cells in the brain that each plays a specialized role in understanding and navigating a 2D world. However, the same brain structures containing these cells play an important role in neural processes that relate to a broader view of a cognitive map such as generalization, inference, imagination, social cognition and memory. They suggest that cognitive maps can be constructed from general models of abstract relationships that are separate from sensory representations and are therefore generalizable across different sensory environments. These abstract representations can be viewed as basic sets for describing relational knowledge. Finally, they speculate that such a view can help understand a number of psychological phenomena, from schemas and generalization to planning and choice.

For Schafer and Schiller (2018), Cognitive maps are encoded in the hippocampal formation and related regions and range from spatial to purely conceptual. Neural mechanisms that encode information in relational structures, up to an arbitrary level of abstraction, can account for such a wide range of representations. Research now indicates that social life can also be mapped by these mechanisms: the spatial location of others, social memory, and even a two-dimensional social space framed by social power and affiliation. Systematically mapping social life onto a relational social space facilitates adaptive social decision making, similar to social navigation. This emerging line of research has implications for research on cognitive mapping, clinical disorders that exhibit hippocampal dysfunction, and the field of social cognitive neuroscience. The hippocampal formation performs functions that include spatial representation and episodic memory. These functions may reflect a multidimensional “cognitive map” that organizes prior experience to support flexible navigation (Tolman, 1948). The discovery of spatially modulated cells in hippocampal and entorhinal cortex led to the belief that these regions encode spatial cognitive maps (Keefe and Nadel, 1978; Eichenbaum and Cohen, 2014). Subsequent research has shown that these regions are also sensitive to a variety of non-spatial and even abstract features, such as sound, time, reward, and concepts (Schiller et al., 2015). The hippocampal formation maps and stores this information in a relational manner, allowing inference and decision making using the stored memory features (Eichenbaum and Cohen, 2014). Emerging research suggests that the hippocampus also represents social stimuli in physical space, information about specific individuals, and abstract social dimensions (Montagrin et al., 2017). The hippocampus and related regions may therefore perform social functions and encode “social space” in the form of a cognitive map. This perspective argues that social cognitive mapping occurs and is supported by mechanisms that map physical space. The argument presents evidence of spatial mapping, followed by evidence that these same mechanisms also map non-spatial and abstract information and allow for the use of cognitive maps in decision making. To support the idea that the hippocampus is involved in social cognitive mapping, they highlight research showing that spatially sensitive hippocampal cells encode social information and discuss how this may relate to the hippocampus’ role in social memory.

Independent of brain area studies and focusing on behavioral studies, Ericson and Warren (2017, 2020) tested in two similar studies (1) the Euclidean hypothesis, a geometrically coherent map; (2) the neighborhood hypothesis, adjacency relationships between spatial regions, based on visible boundaries; (3) the Cognitive graph hypothesis, a network of paths between locations, labeled with approximate local distances and angles. They conclude that primary spatial knowledge is consistent with the cognitive graph hypothesis. Neighborhoods are derived from the graph, and information about local distances and angles is not integrated into a geometrically consistent map. In similar research, Chrastil and Warren (2015), study the structure of spatial knowledge that develops spontaneously during free exploration of a new environment. They present evidence that this structure is similar to a labeled graph, in other words, a network of topological connections between places, labeled with local metric information. In contrast to route knowledge, they find that the most frequent routes and detours to the target locations were not taken during learning. In contrast to purely topological knowledge, participants generally traveled the shortest metric distance to a target, rather than topologically equivalent but longer paths. The results are consistent with the proposition that people learn a labeled graph of their environment.

Finally, Meilinger (2008) in a paper entitled The network of reference frames theory: a synthesis of graphs and cognitive maps, develops in 2008, a network of reference frames theory that explains the orientation behavior of human and non-human animals in directly experienced environmental spaces, such as buildings or cities. This includes self-location, route navigation and flyover navigation. It is a synthesis of graphical representations and cognitive maps, which solves the problems associated with explaining orientation behavior based on either graphs, maps, or both in parallel.

## 3. Conclusion: The cognitive graph–between cognitive map and concept map

To try to understand the seminal opposition between cognitive map and conceptual map covered under the common name of mental map, we first tried to contextualize in broad strokes a set of reflections implied by this opposition. We then focused on examples of urban cognitive map drawings that memorize a route through the city by exemplifying two videos showing the elaboration of this type of representation. One of these videos allowed us to show that a more or less expected representation of the memorization of the traveled space was followed by a much freer representation with a set of abstract and figurative symbols on the back of the sheet. These examples of drawings allowed us to recall certain fundamental characteristics of urban map drawings and to describe the graphic and symbolic elements likely to characterize both the representation of a journey in space (cognitive map) and that of a set of concepts or notions (conceptual map). In accordance with our initial program, we then tried to understand the memorization processes supporting these symbolic processes in these two types of representation by highlighting their complexities and some of their differences. Finally, by summarizing some recent research in neurophysiology and behavioral sciences, we showed that what had seemed to us to be two clearly distinct domains, the cognitive map and the conceptual map, were in fact underpinned by the notion of cognitive graph.

Thus, the graph has appeared with increasing evidence as a common territory to the conceptual map and the cognitive map. It is by considering this notion that we have tried to identify the elements likely to characterize graphical and semantic means that are likely to apply to both concept and cognitive maps. The example of the two videos presented herein has allowed us to exemplify a process called “symbolic projection.” Rather than fully constituted entities that could be described as “symbols,” it is in the dimensional, proportional, proximal, and directional–i.e. so many elements in their graphic elaborations–that the “symbolic projection” terms in the title of this study should be understood. As for “memorial transcription,” we have tried to approach it with a general overview of memory, mainly through the decisive contributions of Tulving (1985) and the relationships between working, episodic and semantic memories. Although episodic memory is often characterized in the WWW (What-Where-When) form, it would perhaps be worthwhile supplementing the more specific form of the concept map or heuristic map in the WWH, (What-Where-How) form.

It is therefore with the notion of cognitive graph that we can group together and put into perspective all the questions raised so far, and particularly the seminal opposition between cognitive and concept maps grouped under the term of mental map. Peer et al. (2021) in their article entitled “Structuring knowledge with cognitive maps and cognitive graphs” show, through studying the functioning of cerebral areas, that each of these maps corresponds to a neuronal ensemble that can correspond to a cognitive map or a graph. Depending on the type of space–landscaped or urban for example–it is sometimes the cognitive graph or the cognitive map that is most appropriate, but more often it is a combination of these two representations types that are used. Furthermore, beyond the types of spaces we move in, the graph can be suitable for “non-spatial” representations such as a social network or a concept map. There is “evidence to suggest that both map-like and graph-like representations exist in the mind/brain, and that they rely on partially overlapping neuronal systems. Maps and graphs can operate simultaneously or separately, and they can be applied to both spatial and non-spatial knowledge. By providing structural frameworks for complex information, cognitive maps and graphs can provide fundamental organizational patterns that enable us to navigate physical, social, and conceptual spaces” (Peer et al., 2021). In a similar way, Behrens et al. (2018) “by describing neuronal parallels between spatial and non-spatial behaviors, have revived the notion of systematic knowledge organization in several domains. They believe that these principles allow for generalizations–abstractions that characterize human cognition. To complete these examples in what is known as spatial and non-spatial knowledge processing, the article by Schafer and Schiller (2018) shows that these general coding mechanisms are able to contextualize the spatial location of others, social memory and even a two-dimensional social space framed by social power and affiliation. In other words, between individual and inter-individual spatial navigation, the localization of others in physical space and the representation of social relations in an abstract network of relations make up the same continuum.

## Ethics statement

Ethical review and approval was not required for the study on human participants in accordance with the local legislation and institutional requirements. Written informed consent for participation was not required for this study in accordance with the national legislation and the institutional requirements.

## Author contributions

The author confirms being the sole contributor of this work and has approved it for publication.

## Footnotes

1 There is a lot of confusion surrounding the terms *carte mentale*, *carte cognitive*, and cognitive map, mind map. In the definitions for mental map and cognitive map that are mainly taken from Wikipedia, there are various and often confusing definitions especially on the French and English language pages. Quote: — “A **cognitive map** (sometimes called, but should not be confused with, a mental map or mental model) is a type of mental representation that serves an individual to acquire, code, store, recall, and decode information about the relative locations and attributes of phenomena in their everyday or metaphorical spatial environment. The concept was introduced by Tolman (1948).” https://en.wikipedia.org/wiki/Cognitive_map. The notion of mental map used here will therefore cover two distinct meanings: (1) cognitive map, (2) concept map. The terms concept map and mind map will be used interchangeably here.

2 “The fact that episodic memory is an active constructive process, leads to the idea that the same cognitive machinery can be used in conscious planning of future actions (Schacter et al., 2007). Indeed, the same neural machinery underlies each process (Buzsáki, 2015). Both episodic recall and planning involve the construction of scenarios starting from some basis of memory and a lot of world knowledge. The only difference is that in one case past events are simulated, while in the other, future events are simulated by a similarly constructive process.” Ware (2020). *Information visualization, perception for design*, Elsevier, p. 412. First published in 2000.

3 They were produced as part of a research project: *Médias situés et mobilités partagées: cartographies mentales, instrumentales et partagées* [Situated media and shared mobilities: mental, instrumental and shared cartographies] 2017–2019, financed as part of the Paris 1 scientific policy, the Collège des Écoles Doctorales and Concordia University in Canada.

4 See footnote *n*°2 in the text that clarifies those different uses of mental maps terms.

5
https://fr.wikipedia.org/wiki/Th%C3%A9orie_des_graphes

6
http://geoconfluences.ens-lyon.fr/glossaire/topologie

7 Types of diagrams, *ArcMap*, https://desktop.arcgis.com/fr/arcmap/10.3/map/graphs/types-of-graphs.htm consulted on 06-22-20.

8 Forty video recordings of the first ANR CORES experiment conducted on 10-24-2020.

9 “Euclidean space: a continuous space defined by reference axes (usually two or three). Locations in a Euclidean space can be specified by coordinates and relationships between locations can be expressed in terms of distances and angles.” Peer et al. (2021) *Ibid.*
